# Design, Synthesis and Biological Evaluation of Ciprofloxacin- Peptide Conjugates as Anticancer Agents

**DOI:** 10.22037/ijpr.2019.111721.13319

**Published:** 2019

**Authors:** Kiana Esfandiari Mazandaran, Sayed Ahmmad Mirshokraee, Khadijeh Didehban, Mohammad Hassan Houshdar Tehrani

**Affiliations:** a *Department of Chemistry, Payam noor University, Tehran, Iran. *; b *Department of Medicinal Chemistry, School of Pharmacy, Shahid Beheshti University of Medical Sciences, Tehran, Iran.*

**Keywords:** Ciprofloxacin- anticancer peptide conjugates, Colon cancer, Breast cancer, Solid Phase Peptide synthesis, MTT test

## Abstract

Cancer has emerged as a leading cause of death throughout the world. Peptides are a novel class of anticancer agents that can specifically target cancer cells with low toxicity to normal tissues and thus, offer new opportunities for future cancer treatment. On the other hand, Ciprofloxacin, an antibiotic, also known to its anticancer property for enabling cell cycle arrest and creating double strand breaks in nucleic acid can trigger apoptosis of cancer cells. Thus, joining anticancer peptides with Ciprofloxacin may be good idea to get benefit of the both compounds’ properties and therefore gives better anticancer agents. The aim of this study was to synthesize Ciprofloxacin- cytotoxic peptide conjugates and to investigate the anticancer activity of the resultant compounds. The conjugates were prepared by solid phase peptide synthesis technique using Fmoc strategy. Anticancer activity of these compounds was examined on three cancer cell lines, HT-29, MCF-7, MDA-MB-231 as well as skin fibroblast cells as a control, employing MTT test. Our results showed that the cytotoxic activity of the synthesized compounds against cancer cells was raised considerably without producing a high toxicity on normal cells. Moreover, Ciprofloxacin-peptide conjugates showed selectivity against different kinds of breast cancer cells, especially on those with triple negative receptors. Therefore, it can be suggested that the strategy of making Ciprofloxacin- peptide conjugates as cytotoxic agents with safety profiles on the normal cells, rise promise to find better chemotherapeutic candidates to combat cancer.

## Introduction

Cancer is considered as one of the most important causes of death globally (1). Conventional chemotherapy for cancer treatment is not very effective because of presenting side effects on normal cells as well as provoking drug resistance by cancer cells (2). Anticancer Peptides (ACPs) are a novel class of anticancer agents which have several favorable characteristics like relatively small size, low immunogenicity as result of occurring naturally, good compatibility in biological systems, many sites of modification in connection with pharmaceutical molecules (3). In addition, ACPs can specifically target cancer cells with low toxicity on normal tissues in usual physiological condition, which will offer new opportunities for cancer prevention and treatment (4-6). 

Fluoroquinolone antibiotics are famous synthetic compounds which show their antibacterial activity by inhibiting two types of bacterial topoisomerase enzymes, i.e., DNA gyrase and topoisomerase IV (7). Inhibition of these enzymes generates and fixes a high level of double-strand DNA cuts within the cells, which ultimately ends to bacterial cell death. Human congener of the DNA gyrase enzyme, called topoisomerase II, does not have affinity enough to quinolones at normally achievable bactericidal dose, and so, these antibiotics do not kill human host cells (8, 9). However, due to the similarities in structural and functional features existing between bacterial DNA gyrase and human topoisomerase II, efforts have been carried out to shift and use bactericidal fluoroquinolones for antitumor activity towards cancer treatment (10, 11). Among fluoroquinolones, ciprofloxacin has been found to be more attractive for this purpose, due to its strong inhibition of topoisomerase II (12). Moreover, a considerable relationship between cytotoxic activity and ability to induce the cleavage of topoisomerase II-DNA complex has been shown for ciprofloxacin (13). So far, ciprofloxacin has been used for its growth inhibitory activity in many cancer cells including bladder, colorectal, and prostate cancer (14-16). In clinical application, ciprofloxacin as an antimicrobial agent is used in low doses and its concentration in serum is not beyond 7µg/ mL (17).It was reported that IC_50_ of ciprofloxacin against DNA gyrase and topoisomerase IV of *E.coli* is 0.34 and 4.6 µg/mL, respectively (18). On the other hand, ciprofloxacin inhibits topoisomerase II activity almost merely in high concentrations, i.e., enzyme inhibition does not occur in low concentrations applications of the drug (19). Moreover, ciprofloxacin as an anticancer agent must be used at higher concentration compared to the amount of its application for treating infections in patient (20). Obviously, using any high concentration of the drugs including antibiotics such as Ciprofloxacin may result to unwanted many side effects in the patient. Therefore, introducing Ciprofloxacin derivatives used as cytotoxic agents with a view of causing less toxicity on normal healthy cells is desirable. This study was designed to use two colorectal anticancer peptides, previously found in common been fractions (21), in conjugating with Ciprofloxacin molecule, to find good anticancer active agents, in a view of achieving benefits from the favorable characteristics of the both anticancer peptides and Ciprofloxacin, combined in a unique molecular identity.

## Experimental

All the chemicals including protected amino acids, Wang resin, and reagents for peptide synthesis were provided by Bachem AG, Switzerland or Santa Cruz Biotechnology Inc; U.S.A. Solvents were purchased from Sigma-Aldrich. Mass spectra of the samples were recorded on an Agilent 6410 QQQ LCMass spectrometer.

*Preparation of* *free Ciprofloxacin (1) *

**Figure 1:**
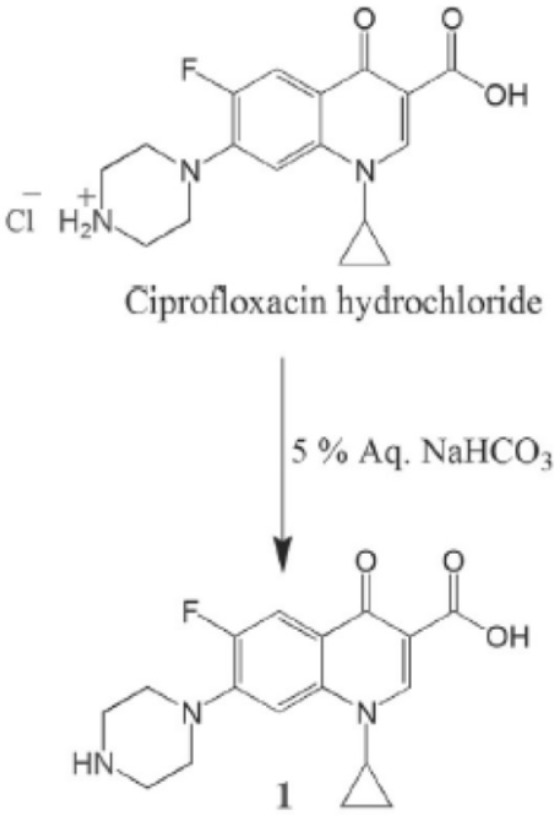
Preparation of free Ciprofloxacin

The ciprofloxacin hydrochloride (5.0 g, 13.59 mmol) was dissolved in water (30 mL) to obtain a clear solution. This solution was treated with an excess of 5% aqueous sodium bicarbonate solution which resulted in the formation of a white precipitate. The precipitate was filtered off and left to be dried as a free ciprofloxacin 1(4.3 g, 12.98 mmol). The free ciprofloxacin was pure enough to be used in coupling with peptides (Figure 1) (22). 

*Preparation of Fmoc- Ciprofloxacin*

**Figure 2:**
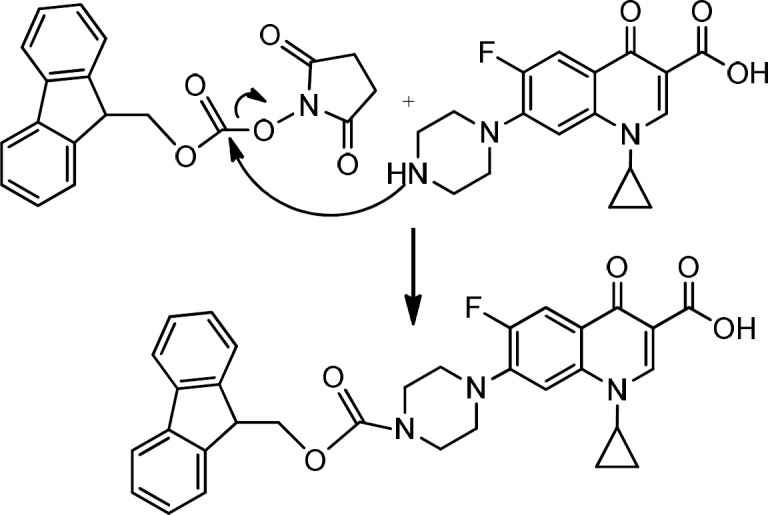
Preparation of Fmoc- Ciprofloxacin

**Figure 3:**
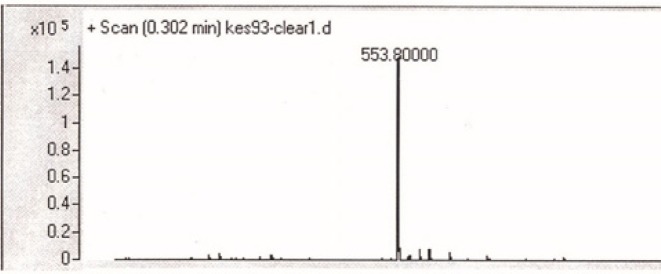
Mass spectrum of Fmoc-Ciprofloxacin

For the *N*- protection of Ciprofloxacin, a published method was used with some modification (23). In brief, free Ciprofloxacin (1 eq) was dispersed in a mixture of water: acetone (50:50) solvent and to this mixture, concentrated sodium carbonate solution was added with stirring until to obtain a clear solution (pH≈11). It was followed by the addition of 9-Fluorenylmethyl *N*-succinimidyl carbonate (Fmoc-OSu) (1.5 eq, dissolved in acetone) to the solution, slowly. The mixture was stirred at room temperature for 1 h. Then, EtOAc was added to the medium, which was acidified with 10% HCl and shaked in a decantor. The organic layer was separated, washed with water several times, dried by sodium sulfate and then precipitated by the addition of petroleum ether. The solid was collected by centrifugation. m/z: 553.80000 (M+1), Yield: 70% (Figures 2, 3).

*Peptide synthesis on resin*

Two peptides, GLTSK, and GEGSGA, previously found in common bean fractions (21) as inhibitors of human colorectal cancer cells, were synthesized by the solid phase peptide synthesis strategy according to the previously reported method using Wang resin (24). 

*Preparation of Fmoc-Ciprofloxacin-peptide conjugate on resin*

Fmoc- Ciprofloxacin (2 meq), HOBt (2 meq), PyBOP (2 meq), were dissolved in a minimum amount of DMF. DIPEA (4 meq) was added to the mixture. The solution was immediately added to the peptidyl resin which was already *N*-terminally deblocked and swollen in DMF:DCM (50:50). The mixture was gently agitated for 3 h, then the solvent was filtered off and the resin was washed with DMF (3 × 5 mL) and DCM (3 × 5 mL). 

*Removing the Fmoc group from Ciprofloxacin-peptide conjugate on resin*

Removing the Fmoc protecting group from the *N*-terminal Ciprofloxacin bound to peptidyl resin was performed by treating the resin with a solution of piperazine/DMF (10%) for 20 min. Then the solution was drained off and the resin was washed with DMF (2 × 2 mL). Chloranil test was used to confirm the result of Fmoc removal by detecting free terminal amine group of ciprofloxacin- peptidyl resin.

*Cleavage of the Ciprofloxacin- peptide conjugate from the resin*

The resin carrying the Ciprofloxacin- peptide conjugate was treated with a solution (10 mL) of trifluoroacetic acid/dichloromethane/anisole /triisopropylsilane (50:45:2.5:2.5) for 2 h and after filtration, the filtrate was precipitated in an ice cold diethyl ether. The precipitated Ciprofloxaxin-peptide conjugate was separated from the etheral solution by filtration, washed with fresh cold ether, and kept in a cold and dark condition. 

*Cell toxicity study*

To determine the cytotoxicity of the two peptides, GLTSK and GEGSGA and their ciprofloxacin conjugates, three human cancer cell lines were employed; MCF-7, MDA-MB-231 (two breast cancer Cell lines), and HT-29 (Human Colorectal Adenocarcinoma Cell Line). Human skin fibroblast cell line was also included for comparison. Cell toxicity experiments were carried out in accordance with the previously reported method (25, 26) with some modification. At 37 °C under CO_2_/air (5:95% )_,_ the cells were grown in RPMI1640 medium, enriched with fetal bovine serum (FBS, 10%), penicillin (100 µg/mL), and streptomycin (100 µg/mL). Cell viability was examined by employing the MTT technique which its principle is on the basis of the transformation of 3-(4, 5-dimethylthiazol-2-yl)-2, 5-diphenyltetrazolium bromide (MTT) dye to formazan crystal by succinate dehydrogenase enzyme of mitochondria in the alive cells. The cells were bred into 96-well plates at a concentration of 10^4^ cells/well and incubated for 24 h. 

The cells were exposed to 10,100 and 1000 µM concentrations of the peptides for 48 h. The supernatant solution was removed and MTT reagent (10 μL, 5 mg/mL in PBS) was added to each well and the microplate was kept at 37°C for 4 h. The medium solution containing MTT was discarded and DMSO (100 μL) was added to each well to dissolve the formazan crystals. The plates were then maintained for 20 min at 37 °C. At the end, the optical density of each well was read at 570 nm against the reference wavelength of 630 nm as the background, employing a spectrophotometer plate reader (Infinite® M200, TECAN) (27). For breast cancer cells, ciprofloxacin as a positive cytotoxic control of the peptides was used. Data were shown as the mean of triplicate measuring of the number of living cells.

## Results

**Figure 4:**
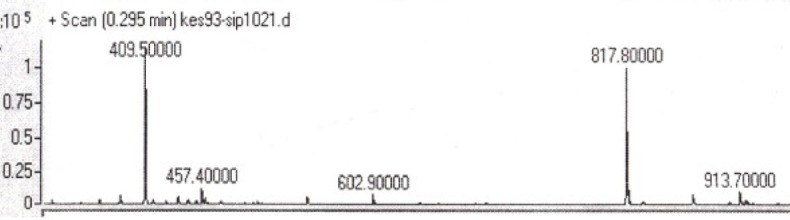
Mass spectrum of Ciprofloxacin-GLTSK peptide conjugate

**Figure 5:**
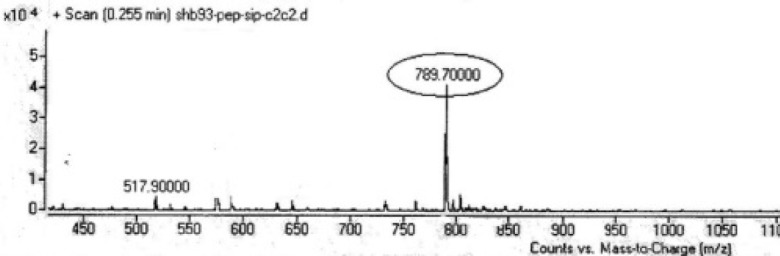
Mass spectrum of Ciprofloxacin-GEGSGA peptide conjugate

**Figure 6:**
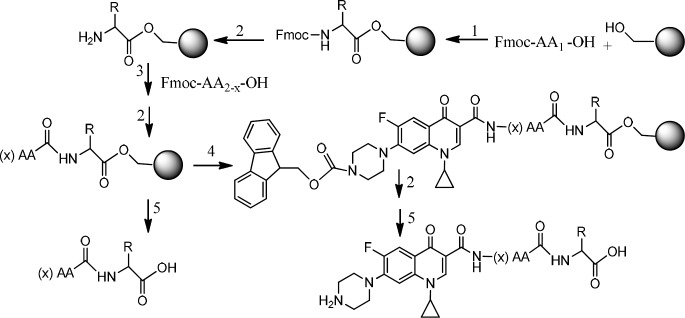
*Preparation of Ciprofloxacin-peptide conjugate *1) HOBt, DMAP, DIC, DMF. 2) Piperazine, DMF.3) HOBt, DIC, DMF.4) Fmoc-ciprofloxacin, PyBOP, DIPEA, DMF.5) TFA with scavengers

Two peptides, GLTSK (C_1_ mother) and GEGSGA (C_2 _mother) were synthesized by solid phase peptide synthesis (SPPS) method using Wang resin with 78% and 75% yields, respectively, and their purities were found good enough according to the mass spectra results . A part of the each peptide, before cleavage from the resin, was also connected *N*-terminally to Fmoc-ciprofloxacin through amide bond formation. After Fmoc removal, the conjugates of Ciprofloxacin-peptides were cleaved from the resin, where the side chain deprotection of the peptides was also happened, simultaneously. Yields were 70-75%. The mass spectra of the desired compounds displayed molecular ion peaks at the appropriate m/z, i.e., 817.80000 (M+1, for Cipro-GLTSK) and 789.70000 (M+1, for Cipro-GEGSGA) (Figures 4, 5). The whole process is shown schematically in Figure 6. Anticancer activities of the C_1_ and C_2_ mother peptides were examined on HT-29 colon cancer cell line and results showed over 90% cell proliferation inhibition using MTT assay (Table 1). All the mother peptides and their Ciprofloxacin conjugates were then exposed to the breast cancer cell lines MCF-7 and MDA-MB-231, as well as the skin fibroblast cells (HFF-1). Ciprofloxacin was used as a reference drug. The results of their anticancer activities, using MTT assay, are given in Table 2.

## Discussion

Before the synthesis of ciprofloxacin- peptide conjugates, it was needed to prepare Fmoc-Ciprofloxacin, since the drug such as an amino acid must be N-terminally blocked in order to avoid byproduct formation during conjugation with peptides. Ciprofloxacin is not so much soluble as a normal amino acid in aqueous reaction medium, even at pH 9-10, and it starts precipitating during reaction with Fmoc-OSu. Therefore, a mixture solution of water (adjusted with pH 11 by saturated sodium bicarbonate solution) and acetone was employed to dissolve ciprofloxacin during a period of 1h time before reaction with Fmoc-OSu. In another experiment, ciprofloxacin HCl was dissolved in water and then acetone was added to the water dropwise, while carefully watching any precipitation does not happen. The solution was adjusted to pH 11 by sodium bicarbonate and then Fmoc-OSu solution (previously dissolved in acetone) was added slowly to the reaction medium while the solution was kept clear.

Many N-terminal derivatives of Ciprofloxacin have been reported to show activities against topoisomerase enzymes and thus were introduced as antibiotic or anticancer agents (28, 29). This is because the structure activity relationship studies recommend that C3- carboxylic acid of cyprofloxacin should be free in order to achieve and preserve biological activity of ciprofloxacin. Nevertheless, few examples of C3- carboxylic acid derivatives have been synthesized which were quite active as antibacterial and anticancer agents (30-32). However, in these Ciprofloxacin derivatives, the chelating sites made by two carbonyl/enol groups at C3 and C4 of Ciprofloxacin structure were reserved, although modified. In the present study, Fmoc-Ciprofloxacin was conjugated with cytotoxic peptides *N*-terminally through the carboxylic acid moiety of Ciprofloxacin, hoping that Ciprofloxacin would keep its chelating property, which along with peptides’ cytotoxicity give higher activity against cancer cells. After conjugation of the drug with peptides attached to the resin, Fmoc removal of the drug-peptidyl resin was performed before cleaving the conjugate from the resin. To do this job, piperazine reagent as a secondary amine base was used. It should be mentioned that the attached piperazine ring, as a part of Ciprofloxacin molecule most probably would not be involved during and after Fmoc group removal in the reaction. That is because the attached piperazine ring was not freely moveable and available in this environment. Moreover, piperazine reagent as a small molecule dissolved in DMF, was used with a concentration very higher than that of piperazine ring as a part of ciprofloxacin molecule bound to a relatively large size peptide molecule attached to the resin. On the other hand, the usual side reaction of the dibenzofulvene molecule, released as the deformed Fmoc group in the reaction, with dissolved piperazine reagent, should not have been occurred with the piperazine ring of the newly conjugate Ciprofloxacin-peptidyl resin, because several on time washing the resin with DMF solvent could not allow such undesired side reaction to occur. Moreover, the conjugated products after cleavage from the resin gave the relevant desired molecular weights of the compounds and not different unwanted molecular weights, as the mass spectra results demonstrated (Figures 4, 5). Anticancer activities of the peptides, GLTSK (C_1_), GEGSGA (C_2 _) and their conjugates examined on HT-29 colon cells showed that high inhibitory activity (around 92%) can be achieved using 10 to 1000 µM concentrations of the corresponding compounds (Table 1). In this respect, concentrations higher than 10 µM did not increase the inhibitory activity of the compounds significantly. This may be interpreted that the efficacy of these compounds for inhibitory action on the aforementioned cells has reached to the maximum with 10 µM concentration and, therefore, with higher concentrations of the compounds it would not raise anymore. The achieved inhibitory action of the peptides was in accordance with the results of anticancer properties of the peptides reported previously (21). In order to examine the cell toxicity of these peptides on other cell lines, two breast cancer cell lines, MCF-7, and MDA-MB-231 were employed to expose to the low concentration (10 µM) of each peptides, a chosen concentration which was obviously more favorable than the other concentrations of the peptides. According to our results shown in Table 2, the both peptides showed cytotoxic effect on the both breast cell lines, and this effect was maintained or intensified by their Ciprofloxacin conjugate on MDA-MB-231 cells. However, C_2_ peptide although showed potent cell toxicity on MCF-7, C_2_Cipro conjugate demonstrated rather weaker toxic effect. Considering the net charges of C_1_ and C_2_ peptides being positive and negative in physiologic pH, respectively and also the C_1_ hydrophobicity being less than C_2_ (21), this phenomenon can be interpreted that MCF-7 cells act more selectively towards allowing toxic compounds to enter their inside, compared to MDA-MB-213 cells, and this selection is in the favor of accepting negative and more hydrophobic ionic molecules (like C_2_ peptide) rather than a zwitterionic (like Ciprofloxacin) and less hydrophobic (like C_2_ Cipro) and positive ionic (like C_1_ peptide) molecules. In this regard, Ciprofloxacin and C_1_ peptide with moderate hydophilicity are more active against MDA-MB-231 cells. Peptides C_1_, C_2_ and their conjugates demonstrated a safety profile on Fibroblast cells (See Table 2). 

**Table 1:** Anticancer activity of pentapeptides GLTSK (C1), hexapeptide GEGSGA (C2), GLTSK- Ciprofloxacin conjugates (C1 Cipro) and GEGSGA –Ciprofloxacin conjugates (C2 Cipro) agents on colon cancer cells

	**Colon Cancer cells (HT-29)** **Concentration (µM)**	**% Inhibition (Mean ±SD)**
C1 mother	10	93.01 ± 1.01
C1 mother	100	93.06 ± 0.48
C1 mother	1000	92.31 ± 0.16
C1 Cipro	10	92.98 ± 21
C1 Cipro	100	93.3 ±.44
C1 Cipro	1000	91.98±.23
C2 mother	10	92.79 ± 0.54
C2 mother	100	93.01 ± 0.57
C2 mother	1000	92.66 ± 0.44
C2 Cipro	10	92.68 ± .44
C2 Cipro	100	92.44 ± .47
C2 Cipro	1000	92.25 ± .44
Ciprofloxacin	10	93.047 ± .44
Ciprofloxacin	100	93± .41
Ciprofloxacin	1000	92.56 ± .41

**Table 2:** Anticancer activity of pentapeptides GLTSK (C1), hexapeptide GEGSGA (C2), GLTSK- Ciprofloxacin conjugates (C1 Cipro) and GEGSGA –Ciprofloxacin conjugates (C2 Cipro) agents on breast cancer cells

**%Inhibition (Mean± SD)**			
**Compounds (Concentration 10µM)**	**MCF-7 cells**	**MDA-MB-231 cells**	**Fibroblast cells**
C1 (mother)	50.11± 2.09	78.7± 1.6	4.41± 2.7
C1 Cipro	73.6± 1.25	82.06± 0.23	3.89± 2.3
C2 (mother)	83.39± 0.84	79.32± 0.23	3.11± 1.19
C2 Cipro	76.84± 0.24	81.16± 0.23	7.27± 0.77
Ciprofloxacin	76.41± 1.08	81.11± 0.23	2.33± 1.19

## Conclusion

Our results demonstrated that Ciprofloxacin derivatives on its carboxylic acid group preserve its anticancer activity. Therefore, Ciprofloxacin-peptide conjugates can be considered as chemotherapy agents for cancer cells, especially the breast cancers containing triple negative receptors on their cells. The strategy of making Ciprofloxacin- peptide conjugates as cytotoxic agents which give safety profiles on the normal cells can make promise to find and introduce the better candidates as new anticancer compounds.
